# Heart Rate Variability in Normotensive and Hypertensive Adults After a Year of Receiving Oxford/AstraZeneca COVID-19 Vaccine: A Cross-Sectional Observational Study

**DOI:** 10.7759/cureus.40010

**Published:** 2023-06-05

**Authors:** Sunita, Manjulata, Himel Mondal, Manish Kumar

**Affiliations:** 1 Physiology, Indira Gandhi Institute of Medical Sciences, Patna, IND; 2 Physiology, All India Institute of Medical Sciences, Patna, IND; 3 Physiology, All India Institute of Medical Sciences, Deoghar, IND

**Keywords:** oxford/astrazeneca covid-19 vaccine, hrv, autonomic function, blood pressure, hypertension, sympathetic, covishield, vaccine, heart rate variability, covid

## Abstract

Background and aim

Heart rate variability (HRV) helps in assessing the autonomic nervous system's function, which has been implicated in cardiovascular disease risk. HRV has been found to be deranged in hypertension. In addition, studies have shown that COVID-19 infection and vaccination can affect HRV. However, the long-term effect of HRV on hypertension has not been explored after COVID-19 vaccination. The objective of this study was to observe the HRV in hypertensive adults after one year of receiving the Oxford/AstraZeneca COVID-19 vaccine and to compare it with normotensive adults.

Methods

The study included 105 normotensives (blood pressure below 120/80 mmHg) and 75 hypertensive participants who had received the Oxford/AstraZeneca COVID-19 vaccine one year prior. HRV was measured using the PowerLab system (ADInstruments) with the participants in a sitting posture. The HRV parameters assessed included the time domain, frequency domain, and nonlinear measures. Data were presented in descriptive and inferential statistical terms, and the parameters of two groups of individuals were compared by either an unpaired t-test or the Mann-Whitney U test.

Results

A total of 105 normotensive participants with a mean age of 42.51 ± 9.28 years and 75 hypertensive participants with a mean age of 44.24 ± 10.19 years comprised the sample (p=0.24). Normotensive individuals had a higher standard deviation of RR intervals, a higher coefficient of variation of RR intervals, a higher standard deviation of heart rate, and a higher percentage of successive differences in RR intervals in the time domain. They also had higher values of very low-frequency power, low-frequency (LF) power, and high-frequency (HF) power in the frequency domain. The LF/HF ratio was not significantly different between the two groups. In nonlinear analysis, SD2, a measure of long-term heart rate variability, was higher in normotensive individuals.

Conclusion

The Oxford/AstraZeneca COVID-19 vaccine did not have a significant effect on HRV parameters in normotensive and hypertensive adults one year after vaccination. However, changes in HRV parameters were observed between supine and standing positions, suggesting the importance of postural changes in HRV assessment.

## Introduction

Heart rate variability (HRV) measures beat-to-beat changes in the heart rate that occur in response to internal and external stimuli [1]. It is a noninvasive measure of autonomic nervous system activity. It has been used as an indicator of a range of health conditions. In healthy individuals, HRV tends to be higher, reflecting the adaptability and flexibility of the autonomic nervous system to respond to various stimuli. Conversely, reduced HRV is associated with physiological and pathological conditions such as cardiovascular diseases, diabetes, stress, and sleep disorders [2]. Monitoring HRV can provide insights into an individual's overall health status as well as their resilience and ability to cope with physiological and psychological challenges. Furthermore, HRV analysis has emerged as a promising tool in the field of preventive medicine, offering early detection and risk assessment for various health conditions [3].

The COVID-19 pandemic has affected the global health system in various aspects, with the majority of the population being either infected or vaccinated. The virus primarily affects the respiratory system, but it can also lead to inflammation throughout the body, including the heart muscle [4,5]. The COVID-19 infection can result in myocarditis, which can cause an elevated heart rate in some patients. The development of myocarditis in individuals with COVID-19 infection is believed to involve direct damage to the heart muscle cells by the virus itself. Additionally, it is suggested that there might be a contribution from the cytokine storm. Similarly, the mechanisms underlying myocarditis associated with COVID-19 vaccines encompass various factors such as molecular mimicry, the formation of autoantibodies, immune reactivity to messenger ribonucleic acid (mRNA), activation of preexisting immune dysfunctions, and genetic predisposition [6]. However, not all COVID-19 patients experience myocarditis. The COVID-19 vaccination can also lead to changes in heart rate, including a temporary increase as a result of the body's immune response [7].

Hypertension is a common condition that affects millions of people worldwide. In 2019, the worldwide prevalence of hypertension among adults aged 30 to 79 was 32% for women and 34% for men, which is comparable to the rates observed in 1990, where women had a prevalence of 32% and men had a prevalence of 32% [8]. The condition is characterized by elevated blood pressure levels, which can lead to serious health complications if left untreated. Patients with hypertension have a lower HRV compared to normotensive individuals. Reduced HRV in hypertensive individuals has been attributed to increased sympathetic nervous system (SNS) activity and reduced parasympathetic nervous system (PNS) activity, which is characteristic of hypertension [9]. Furthermore, it has been suggested that HRV can be used as a prognostic marker for the development of hypertension-related complications, such as myocardial infarction, stroke, and heart failure [10].

India has been administering the Oxford/AstraZeneca COVID-19 vaccine, also known as the Covishield vaccine, as part of its national COVID-19 vaccination program [11]. The HRV may be deranged immediately after vaccination and may return to pre-vaccination status after a few days [12]. The decrease in HRV was associated with the production of specific antibodies against the SARS-CoV-2 spike protein, and this is a temporary phenomenon [13]. However, to date, no study has been conducted in India to observe the long-term changes in HRV in hypertensive patients after COVID-19 vaccination.

With this background, we aimed to conduct this study to find the comparative HRV in normotensive and hypertensive adults after one year of receiving the Oxford/AstraZeneca COVID-19 vaccine. The result of the study would highlight any possible derangements of HRV in hypertensive individuals in comparison to normotensive individuals in the long run after the vaccination.

## Materials and methods

Study design and setting

This is a cross-sectional observational study that started on March 15, 2022 and ended on May 15, 2022. The study was conducted at the Indira Gandhi Institute of Medical Science in Patna, India. This teaching hospital is situated in the eastern part of India.

Ethics

This study involved adult (age >18 years) human research participants. According to the guidelines in the country, any adult can provide consent for voluntary participation in research. We have obtained written informed consent in the local language from all the participants. We further declare that we followed the guidelines laid out in the Declaration of Helsinki, updated in 2013. This study was approved by the institutional ethics committee (reference number: 342/IEC/IGIMS/2021, dated December 13, 2021).

Research participants

**Figure 1:**
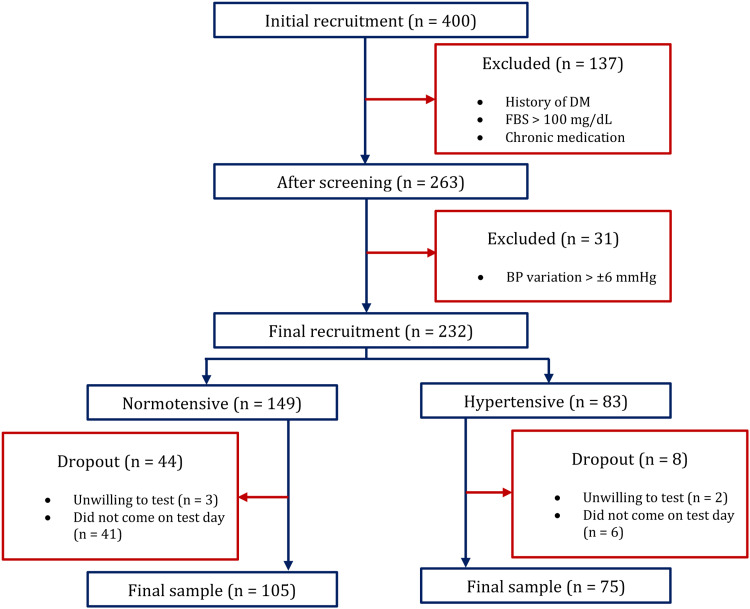
Recruitment of participants n: number; DM: diabetes mellitus; FBS: fasting blood sugar; BP: blood pressure

We recruited a hospital-based convenience sample from apparently healthy people who came to the hospital accompanying patients. We approached the potential participants with research briefs in the local language. Any subject with an age above 18 who had two doses of the Oxford/AstraZeneca COVID-19 vaccine 52 weeks prior was recruited for the study. In the pool, anyone with any acute or chronic disease except hypertension was excluded. The recruitment of participants is shown in Figure 1.

The blood pressure was measured by an aneroid sphygmomanometer (with ± 2 mm Hg accuracy) and an analog stethoscope by the auscultatory method by three expert physicians with more than five years of experience. The highest reading was taken as the final reading. However, where there was more than ± 6 mm Hg difference between the two readings, the participant’s data were not included in the analysis. We used normal systolic blood pressure to be below 120 mmHg and diastolic blood pressure to be below 80 mmHg. The criteria used to consider the participants as hypertensive were according to the criteria of the American Heart Association [14]. Blood sugar was measured from a blood sample taken from the antecubital vein and collected in a commercial vacutainer with an anticoagulant. The sample was immediately transferred to the central laboratory and tested for plasma glucose by an automated analyzer using the glucose oxidase peroxidase method. We also recorded the age, sex, and weight (on a digital weighing scale with an accuracy of 100 g) of the participants.

After that, patients were sent for an autonomic function test. The physicians who measured the autonomic function tests were not aware of the blood pressure of the participants.

Measurements

**Figure 2:**
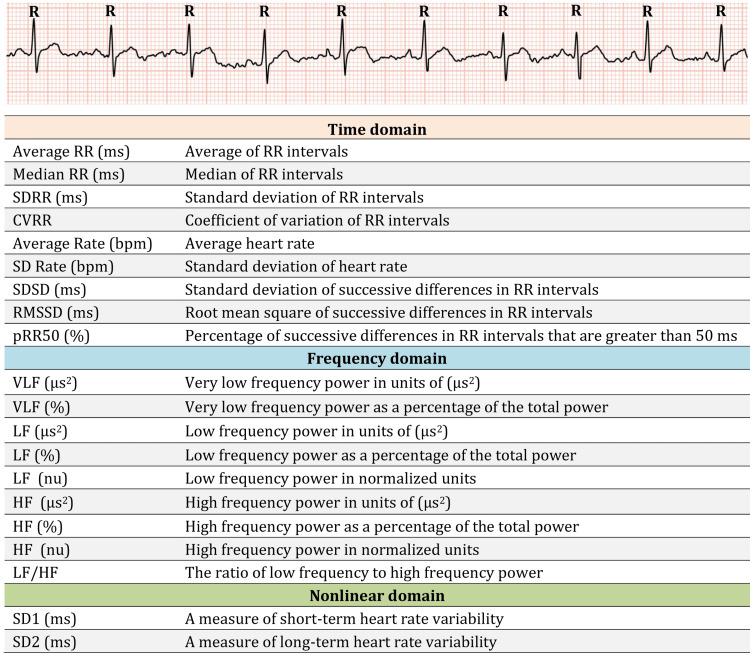
Heart rate variability parameters measured in this study

HRV was recorded by an automated instrument, the ML818 PowerLab 15T (ADInstruments Pty Ltd, Australia), and its paired software for Windows, LabChart Pro 8 (version 8.1.13.12) [15]. The test parameters are shown in Figure 2.

A single experienced physician measured the HRV of all the participants. All the tests were conducted in a controlled environment of 24°C with 50% humidity. All the participants were instructed not to exercise or engage in moderate-to-severe physical activity in the last 24 hours, to avoid alcohol or caffeine in the last 12 hours, and to skip the morning dose of any medication for hypertension that may affect heart rate, like beta blockers. The test was demonstrated to the patients before starting the procedure, and the first test was carried out to make the subject familiar with the instrument. Then the actual test was carried out. Both procedures were conducted in a sitting posture. A same-sex attendant and laboratory technicians were helping with the recording.

Statistical analysis

The data were saved in Microsoft Excel 2010 and expressed as central tendencies after checking the normal distribution. The HRV and other parameters were compared by either an unpaired t-test or a Mann-Whitney U test according to the normality of the data. Statistical analyses were conducted in GraphPad Prism 7 (GraphPad Software, USA).

## Results

A total of 105 normotensive participants (male: 66, female: 39) with a mean age of 42.51 ± 19.28 years and 75 hypertensive participants (male: 59, female: 16) with a mean age of 44.24 ± 10.19 years comprised the two groups in the study. The age, weight, resting heart rate, and blood pressure are shown in Table 1.

**Table 1:** Distribution of normotensive and hypertensive study participants *Chi-square test p-value; †unpaired t-test p-value

Parameter	Normotensive (n = 105)	Hypertensive (n = 75)	p-value
Sex (male : female)	66:39	59:16	0.02*
Age (years)	42.51±19.28	44.24±10.19	0.24
Weight (kg)	63.78±12.87	64.74±13.97	0.63
Resting heart rate (bpm)	74.53±8.15	73.23±7.44	0.28
Systolic blood pressure (mm Hg)	110.34±5.83	134.45±7.25	<0.0001†
Diastolic blood pressure (mm Hg)	74.19±3.17	90.99±6.43	<0.0001†

The time-domain comparative data in the two groups are shown in Table 2. There were significant differences between the two groups for the standard deviation of RR intervals (SDRR), the coefficient of variation of RR intervals (CVRR), the standard deviation of heart rate (SD rate), and the percentage of successive differences in RR intervals that are greater than 50 ms (pRR50), with higher values in normotensive individuals.

**Table 2:** Time-domain parameters of heart rate variability in normotensive and hypertensive participants *Statistically significant p-value of the Mann-Whitney U test Average RR: average of RR intervals; median RR: median of RR intervals; SDRR: standard deviation of RR intervals; CVRR: coefficient of variation of RR intervals; average rate: average heart rate; SD rate: standard deviation of heart rate; SDSD: standard deviation of successive differences in RR intervals; RMSSD: root mean square of successive differences in RR intervals; pRR50: percentage of successive differences in RR intervals that are greater than 50 ms

Parameters	Normotensive (n = 125)	Hypertensive (n = 75)	p-value
	Median (first quartile – third quartile)		
Average RR (ms)	733.3 (655.9 - 789.7)	704.1(619.25- 785.85)	0.29
Median RR (ms)	733 (651 - 794)	705 (632.5 - 784)	0.35
SDRR (ms)	38.46 (23.79 - 48.28)	26.67 (21.08- 36.58)	0.005*
CVRR	0.053 (0.034 - 0.073)	0.039 (0.027 - 0.049)	0.0001*
Average Rate (bpm)	81.79 (75.66 - 91.48)	81.01 (71.09- 93.46)	0.67
SD Rate (bpm)	4.31 (2.68 - 5.59)	2.92 (1.95 – 4.08)	0.0003*
SDSD (ms)	27.24 (15.46 - 41.35)	21.25 (12.49 – 36.21)	0.124
RMSSD (ms)	27.22 (15.46 - 42.01)	21.23(12.48 – 36.03)	0.1
pRR50 (%)	4.3 (0.49 - 16.46)	0.85 (0.21 – 5.3)	0.007*

The frequency-domain comparative data for the two groups are shown in Table 3. Normotensive individuals had higher values of very low-frequency (VLF) power, low-frequency (LF) power, and high-frequency (HF) power than hypertensive individuals. The LF/HF ratio was not significantly different between the two groups. Interestingly, the percentage of VLF and HF was not significantly different between the two groups.

**Table 3:** Frequency-domain parameters of heart rate variability in normotensive and hypertensive participants *Statistically significant p-value of the Mann-Whitney U test VLF (μs2): very low-frequency power in units of (μs2); VLF (%): very low-frequency power as a percentage of the total power; LF (μs2): low-frequency power in units of (μs2); LF (%): low-frequency power as a percentage of the total power; LF (nu): low-frequency power in normalized units; HF (μs2): high-frequency power in units of (μs2); HF (%): high-frequency power as a percentage of the total power; HF (nu): high-frequency power in normalized units; LF/HF: the ratio of low-frequency to high-frequency power

Parameters	Normotensive (n = 125)	Hypertensive (n = 75)	p-value
	Median (first quartile – third quartile)		
VLF(μs^2^)	353.9 (149.3 - 757.3)	209.8 (93.49 - 545.85)	0.008*
VLF (%)	40.92 (27.41 - 56.95)	41.64 (21.43 - 60.41)	0.87
LF(μs^2^)	224.4 (98.17 - 481.1)	147.7 (46.71 - 289.9)	0.03*
LF (%)	23.32 (16.85 - 33.31)	23.65 (15.61 - 31.21)	0.62
LF (nu)	43.25 (31.75 - 59.01)	46.46 (28.54 - 61.48)	0.02*
HF(μs^2^)	307.1 (85.84 - 732.8)	136.6 (46.48 - 394.45)	0.008*
HF (%)	29.35 (17.06 - 41.59)	26.81 (16.66 - 49.11)	0.89
HF (nu)	55.49 (37.91 - 65.05)	51.4 (38.34 - 63.12)	0.58
LF/HF	0.78 (0.49 - 1.53)	0.89 (0.45 - 1.6)	0.69

Nonlinear parameters between the two groups are shown in Table 4. There was a significant difference in the measure of short-term heart rate variability (SD2) between the two groups, with normotensive individuals having higher values than hypertensive individuals. However, there was no significant difference in the measure of long-term heart rate variability (SD1) between the two groups.

**Table 4:** Nonlinear parameters of heart rate variability in normotensive and hypertensive participants *Statistically significant p-value of the Mann-Whitney U test SD1: a measure of short-term heart rate variability; SD2: a measure of long-term heart rate variability

Parameters	Normotensive (n = 125)	Hypertensive (n = 75)	p-value
	Median (first quartile – third quartile)		
SD1 (ms)	19.8 (10.93 - 29.24)	15.02 (8.84 - 26.42)	0.18
SD2 (ms)	46.93 (29.63 - 60.64)	32.49 (24.5 - 46.68)	0.002*

## Discussion

The study suggests that hypertensive individuals have reduced HRV compared to normotensive individuals in the time domain, as evidenced by lower SDRR, CVRR, SD rate, and pRR50 values. However, other HRV parameters, such as the RMSSD and SDSD, did not show significant differences between the two groups. In hypertension, sympathetic activity is increased and parasympathetic activity is decreased, leading to a decrease in the overall variability of RR intervals. This reduction in HRV is associated with an increased risk of adverse cardiovascular events in patients with hypertension [16]. This pattern is prevalent after one year of COVID-19 vaccination.

Similar to the time domain, in the frequency domain, we found that hypertensive individuals had significantly lower SDRR and CVRR values, indicating reduced HRV compared to normotensive individuals. The HF values were significantly higher in normotensive individuals, indicating increased parasympathetic activity. The LF/HF ratio did not differ significantly between the groups. These findings suggest that hypertension is associated with reduced HRV and altered autonomic activity [17].

In non-linear parameters, the results showed that SD1, which reflects short-term variability, was not significantly different between the two groups. However, the SD2, which reflects long-term variability, was significantly lower in hypertensive individuals compared to normotensive individuals. This suggests that hypertensive individuals have reduced long-term HRV compared to normotensive individuals, which may be indicative of decreased parasympathetic modulation in hypertensive individuals [9].

There is a significant body of literature suggesting that hypertension is associated with reduced HRV. Several studies have reported that hypertensive individuals have lower values of time-domain HRV parameters. Some studies have also suggested that the LF/HF ratio, which is an indicator of sympathovagal balance, is increased in hypertensive individuals [18, 19, 20]. However, we found no difference in the LF/HF ratio between normotensive and hypertensive individuals. Overall, our study supports the finding and establishes that even after a long time of receiving the COVID vaccine, the difference in HRV between normotensive and hypertensive patients remains the same as in the pre-COVID era.

The current study has some limitations that should be considered when interpreting the results. Firstly, the sample size of the hypertensive group was relatively small, which may limit the generalizability of the findings. Secondly, the study did not control for confounding factors that may influence HRV, such as physical activity and other undetected comorbidities. Thirdly, the study only measured HRV at a single time point, and it is unclear whether the results would be consistent over time or in response to different physiological or environmental conditions. Lastly, this finding is limited to only vaccinated people who received the COVISHIELD® vaccine. In India, another vaccine, COVAXIN®, is available. However, when we conducted the study, the area of the study had very few people vaccinated with COVAXIN®. Hence, we did not include those participants. In any future study, a comparative study can be conducted to observe if any difference in HRV is present among recipients of different vaccines.

## Conclusions

The study compared heart rate variability (HRV) between normotensive and hypertensive participants after a year of receiving the Oxford/AstraZeneca COVID-19 vaccine. The hypertensive group showed significantly lower values for some HRV parameters, indicating reduced parasympathetic activity and increased sympathetic activity. These findings suggest that hypertension may be associated with autonomic dysfunction. Hence, there are no significant changes in the pattern of HRV that is prevalent in the literature on autonomic functions among normotensive and hypertensive individuals. However, more research is needed to confirm these results in the future.
